# From mono- to bivalent: improving theranostic properties of target modules for redirection of UniCAR T cells against EGFR-expressing tumor cells *in vitro* and *in vivo*

**DOI:** 10.18632/oncotarget.25390

**Published:** 2018-05-22

**Authors:** Susann Albert, Claudia Arndt, Stefanie Koristka, Nicole Berndt, Ralf Bergmann, Anja Feldmann, Marc Schmitz, Jens Pietzsch, Jörg Steinbach, Michael Bachmann

**Affiliations:** ^1^ UniversityCancerCenter (UCC) Dresden, Tumor Immunology, ‘Carl Gustav Carus’ Technische Universität Dresden, Dresden, Germany; ^2^ Helmholtz-Zentrum Dresden-Rossendorf (HZDR), Institute of Radiopharmaceutical Cancer Research, Dresden, Germany; ^3^ German Cancer Consortium (DKTK), part\ner site Dresden and German Cancer Research Center (DKFZ), Heidelberg, Germany; ^4^ Institute of Immunology, Medical Faculty, ‘Carl Gustav Carus’ Technische Universität Dresden, Dresden, Germany; ^5^ National Center for Tumor Diseases (NCT), partner site Dresden, Dresden, Germany; ^6^ Faculty of Chemistry and Food Chemistry, School of Science, Technische Universität Dresden, Germany

**Keywords:** CAR T cell immunotherapy, solid epithelial tumor, nanobody, affinity, PET imaging

## Abstract

CAR-modified T cells show impressive results in clinical trials. However, cytokine release syndrome and “on-target, off-tumor” reactions represent most concerning side effects. To improve the safety of CAR-T cell therapy, we established a switchable CAR platform termed UniCAR system consisting of two components: UniCAR-modified T cells and tumor-specific target modules (TM). For treatment of EGFR^+^ epithelial tumors, we recently described a monovalent nanobody-based α-EGFR TM, either expressed in bacteria or eukaryotic cells. In spite of the identical primary sequence the eukaryotic TM showed a reduced killing capability and affinity. Here we describe a novel bivalent α-EGFR-EGFR TM. As expected, the avidity of the bivalent TM is higher than that of its monovalent counterpart. Binding of neither the monovalent α-EGFR TM nor the bivalent α-EGFR-EGFR TM to EGFR effected the EGF-mediated signaling. While the monovalent α-EGFR TM could only mediate the killing of tumor cells expressing high levels of EGFR, the bivalent α-EGFR-EGFR TM could redirect UniCAR T cells to tumor cells expressing low levels of EGFR. According to PET experiments *in vivo*, the increased avidity of the bivalent α-EGFR-EGFR TM improves the enrichment at the tumor site and its use for PET imaging.

## INTRODUCTION

The epidermal growth factor receptor (EGFR) is a ubiquitously expressed transmembrane protein of the HER family of tyrosine kinase receptors that plays a central role for normal cell and organ development [1–3]. Ligand binding activates downstream signaling cascades involved in controlling cell proliferation and differentiation, apoptosis, cell migration as well as angiogenesis [4–6]. Even minor disruptions in the EGFR signaling pathway can promote tumor growth [3]. Hence, many cancers of epithelial origin, e.g. head and neck, colorectal and lung tumors, are characterized by EGFR overexpression [1–3, 7] or mutated EGFR forms [3, 8, 9]. High EGFR expression level correlates with poor prognosis [10] and an increased resistance to chemotherapy and radiation [11–15]. This underlines the potential of EGFR as a promising target for cancer immunotherapy. Within the last two decades, many EGFR-targeted therapies have emerged. These include tyrosine kinase inhibitors (Erlotinib, Gefitinib, Afatinib) and α-EGFR monoclonal antibodies (Cetuximab, Panitumumab, Necitumumab) approved for treatment of metastatic colorectal cancer, head and neck cancer, pancreatic cancer and lung cancer [3, 16–18]. Nonetheless, as learned from both preclinical and clinical studies therapeutic approaches are often accompanied by mild to severe side effects due to the widespread EGFR expression on healthy tissues [19, 20]. This clearly emphasizes the urgent need for a more precise control of highly effective EGFR directed cancer treatments.

**Figure 1:**
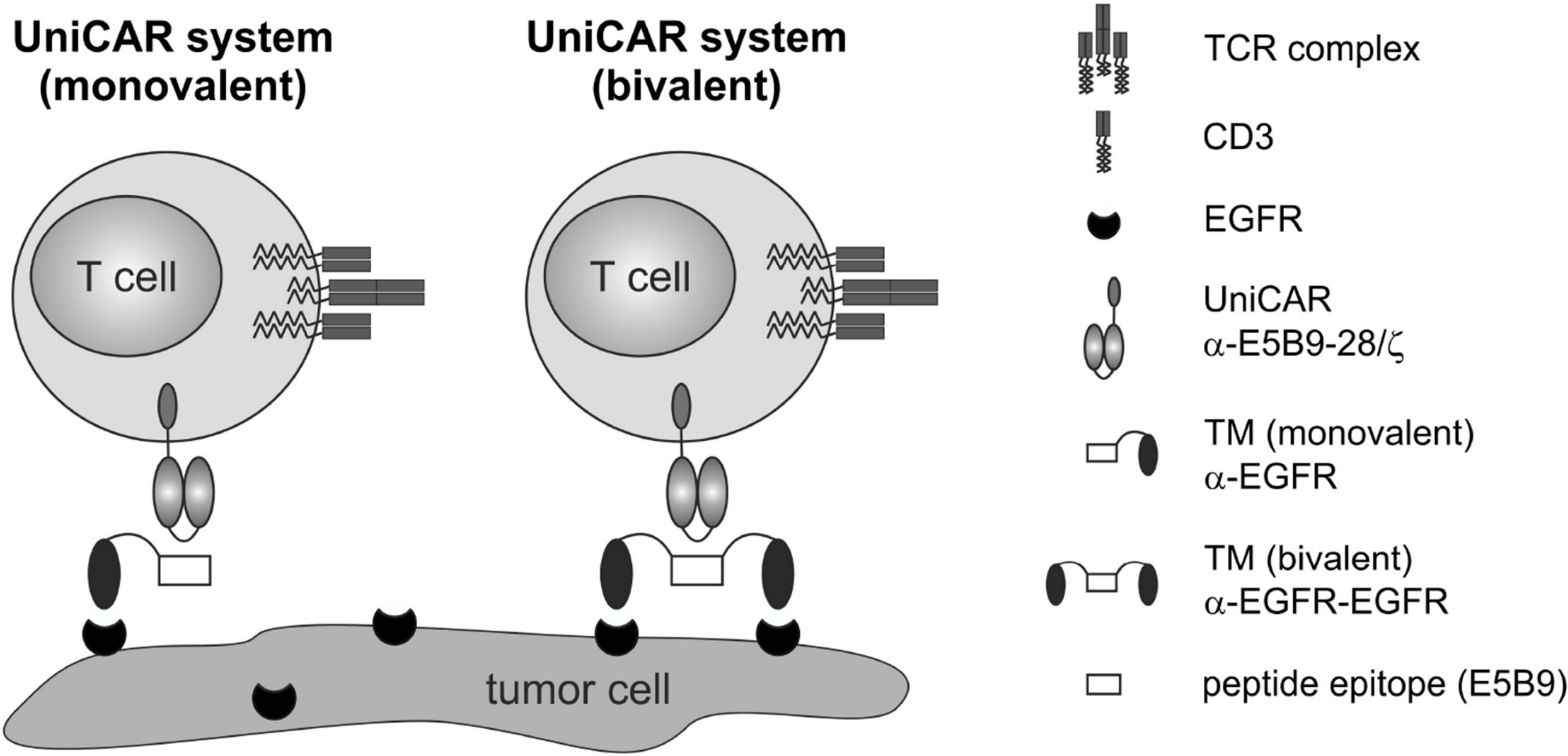
Redirection of UniCAR-armed T cells via EGFR-specific target modules The UniCAR system is based on two distinct components, the universal, signal-transducing UniCAR and a replaceable, tumor-specific target module (TM). By simultaneous binding of the tumor-associated antigen (here EGFR) and the α-E5B9 single-chain fragment variable of the UniCAR, E5B9-tagged TMs are able to mediate a cross-linkage of T cells and tumor cells. For an EGFR-specific targeting, the TMs consist of either one (left panel, monovalent) or two nanobody domains (right panel, bivalent) with specificity for EGFR.

Recently, we described the modular UniCAR platform technology for retargeting of T cells to various tumor-associated surface antigens (TAAs) including EGFR that meets this criterion [21–27]. It represents a unique chimeric antigen receptor (CAR) T cell therapy combining T cells engrafted with a universal CAR (UniCAR) and an antibody (Ab)-based component referred to as target module (TM) (Figure 1). Like conventional CARs [28, 29], the UniCAR construct consists of an extracellular Ab-derived binding moiety, a transmembrane domain and intracellular activation motifs derived from CD3ζ and CD28 for signal transduction. In contrast to CARs, the extracellular binding domain of UniCARs does not recognize a certain TAA on tumor cells but the short peptide epitope E5B9 (UniCAR epitope) used to tag the TM. Thus, UniCAR-modified T cells are per se inactive. The TM in turn confers the UniCAR system its tumor specificity. Due to its composition of a binding moiety for a TAA and the E5B9-tag it acts as a key link between UniCAR-armed T cells and target-positive cancer cells (Figure 1). Despite this modular character, tumor cell elimination is mediated with a high specificity and effectiveness similar to conventional CAR approaches [e.g. 24]. Beyond that, after infusion of UniCAR-engrafted T cells into patients effector mechanisms are only turned on by administration of tumor-specific TMs and inversely turned off by interruption of TM supply, which represents the main advantage of the UniCAR system. It provides an important self-limiting safety switch to quickly reduce or control critical side effects often accompanied by conventional CAR T cell therapies: (I) cytokine release syndrome (CRS) as a result of excessive “on-target, on-tumor” toxicity [30–32] and (II) elimination of healthy cells due to uncontrollable “on-target, off-tumor” effects [31–33]. Currently, alleviation of these partly life-threatening side effects requires further treatment with other immunomodulating drugs including corticosteroids, interleukin-6 receptor blockade (Tocilizumab) and immunoglobulins [31, 32, 34, 35]. This should not be necessary using the UniCAR system as it enables a direct and precise control of T cell activity.

Considering that EGFR is a widespread tumor marker also expressed on normal epithelial cells, the UniCAR technology might be an appropriate solution for efficient EGFR-targeted therapy that simultaneously provides the necessary control mechanisms. As proof-of-concept, we recently reported on UniCAR-armed T cells successfully redirected to EGFR^+^ tumors *in vitro* and *in vivo* via a novel nanobody (Nb)-based α-EGFR TM expressed in *E. coli* (termed α-EGFR TM (pro)) or eukaryotic CHO cells (termed α-EGFR TM) [23]. Pharmacokinetic studies in immunodeficient mice revealed that TMs can be released from UniCAR-TM complexes and thereby support the idea of the on/off-switchable UniCAR system. For an unknown reason, the α-EGFR TM (pro) showed not only an overall enhanced functionality in comparison to the eukaryotic one but also a higher affinity. We therefore asked whether or not we can further improve the effectiveness of α-EGFR TMs by increasing their binding affinity. To answer this question, we constructed a novel bivalent α-EGFR-EGFR TM by fusion of two α-EGFR Nb domains via the E5B9-tag. After expression in CHO cells, its binding avidity, potential EGFR-mediated signaling effects, anti-tumor efficiency and pharmacokinetic behavior were compared to the previously described monovalent α-EGFR TM.

Here we report that the enhanced avidity of the bivalent α-EGFR-EGFR TM improves both its killing capability and its use as PET tracer. Neither the monovalent nor the bivalent TM mediates EGFR signaling under retargeting conditions. We also show that the binding capability of the TM in combination with the density of EGFR on the tumor cell decides whether or not UniCAR T cells will attack the target cell.

## RESULTS

### Establishment of a novel bivalent EGFR-specific TM

For arming the modular UniCAR platform, we established a novel bivalent TM for redirection of UniCAR T cells against EGFR^+^ carcinoma cells (Figure 1). So far, a monovalent α-EGFR TM has been successfully generated and characterized [23]. However, the chosen expression system (eukaryotic vs. prokaryotic) influenced its affinity and functionality within the UniCAR system [23]. To elucidate whether TM functionality can be further improved by an increase in affinity, we here performed comparative analyses between monovalent and bivalent EGFR-specific TMs both expressed in CHO cells.

**Figure 2:**
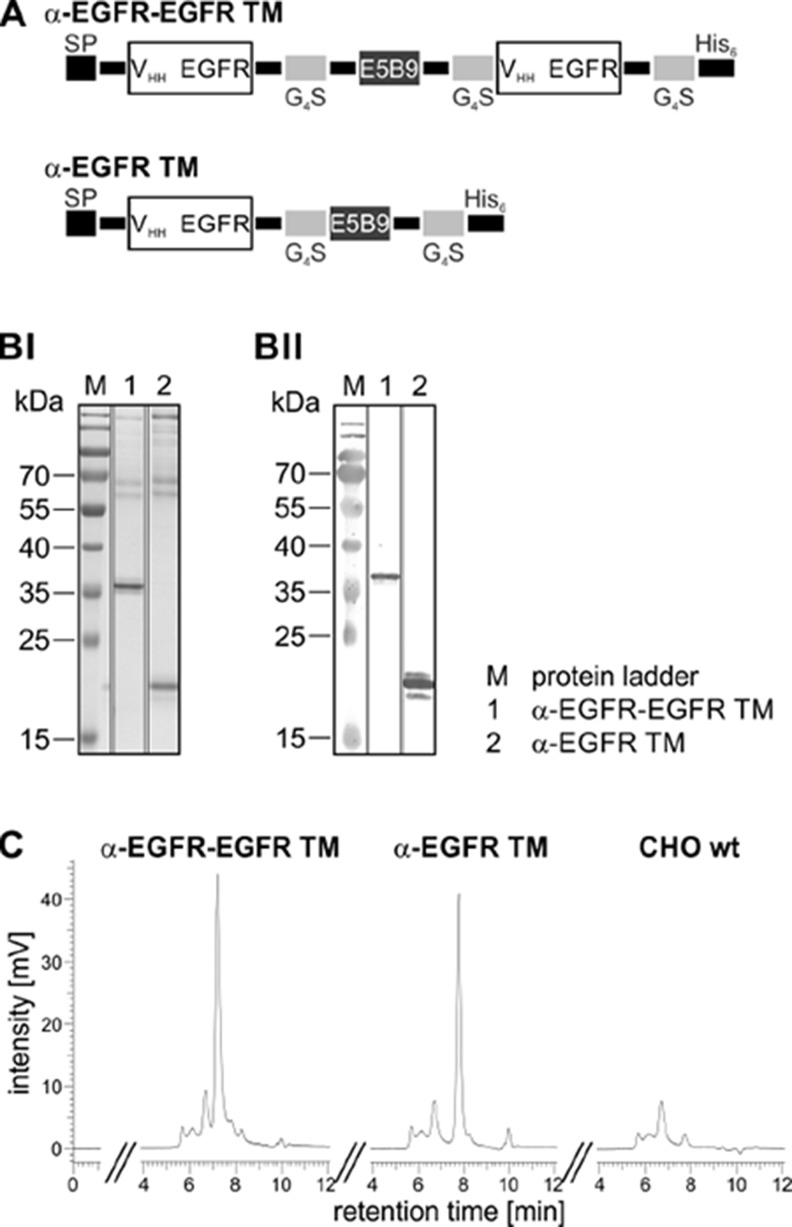
Biochemical characterization of the mono- and bivalent EGFR-specific TM (**A**) The α-EGFR-EGFR TM consists of two camelid Ab-derived α-EGFR(7C12) nanobody domains (V_HH_) separated via the E5B9-tag while the monovalent α-EGFR TM contains a single nanobody domain. The recombinant Abs are further equipped C-terminally with six histidine residues (His_6_) for protein purification and detection. To ensure Ab secretion, the constructs are additionally endowed N-terminally with a signal peptide (SP). (B) After eukaryotic expression in CHO cells, the EGFR-specific TMs were purified by Ni-NTA affinity chromatography. The elution fractions of the α-EGFR-EGFR TM (lane 1) and α-EGFR TM (lane 2) were separated via SDS-PAGE and (**BI**) subsequently stained with Coomassie Brilliant Blue G250 or (**BII**) transferred onto nitrocellulose membranes to detect recombinant proteins via their C-terminal His_6_-tag. M, molecular weight marker. (**C**) To further analyze the mono- and bivalent TM, 15 µg of the respective elution fraction and 15 µl of purified CHO wt supernatant were analyzed by size exclusion chromatography.

As schematically summarized in Figure 2A, the bivalent α-EGFR-EGFR TM was generated by flanking the UniCAR epitope with one EGFR-specific camelid Nb-domain (clone 7C12) [36] on each side. The recently described monovalent α-EGFR TM contains only one Nb-domain C-terminally equipped with the UniCAR epitope. At the N-terminus, both TMs contain the same signal peptide for triggering secretion into cell culture supernatant. They further comprise a C-terminal histidine (His_6_)-tag for protein purification and detection. The different domains of the recombinant Ab molecules were fused via flexible peptide linkers consisting of glycine and serine residues (G_4_S).

After expression by a permanent Ab-producing CHO cell line the recombinant proteins were isolated from cell culture supernatant via Ni-NTA affinity chromatography. For biochemical characterization, the EGFR-specific TMs were analyzed by SDS-PAGE (Figure 2BI) and immunoblotting (Figure 2BII). The results confirm that both constructs were successfully expressed as full-length proteins and can be detected via their C-terminal His_6_-tag. By comparing the molecular weight of 36 kDa with the theoretically calculated size of 32 kDa it becomes obvious that the bivalent TM exhibits a slightly aberrant mobility which may be caused by posttranslational modifications or insufficient cleavage of the N-terminal signal peptide. As already seen by SDS-PAGE (Figure 2BI) and also confirmed by HPLC size exclusion chromatography (Figure 2C), the eluates mainly contain the respective TMs (α-EGFR-EGFR TM: 72% purity, α-EGFR TM: 69% purity) but also high molecular weight (HMW) proteins. Similar contaminations were already detected in preparations of other TMs after isolation from culture supernatants of CHO cells [23–25]. Moreover, the same HMW proteins are co-purified from cell culture supernatant of CHO wildtype (wt) cells lacking any expression vectors (Figure 2C, CHO wt). Therefore, these co-isolated HMW proteins represent most probably proteins from CHO cell culture supernatants.

### Binding analysis of the novel bivalent α-EGFR-EGFR TM

**Figure 3:**
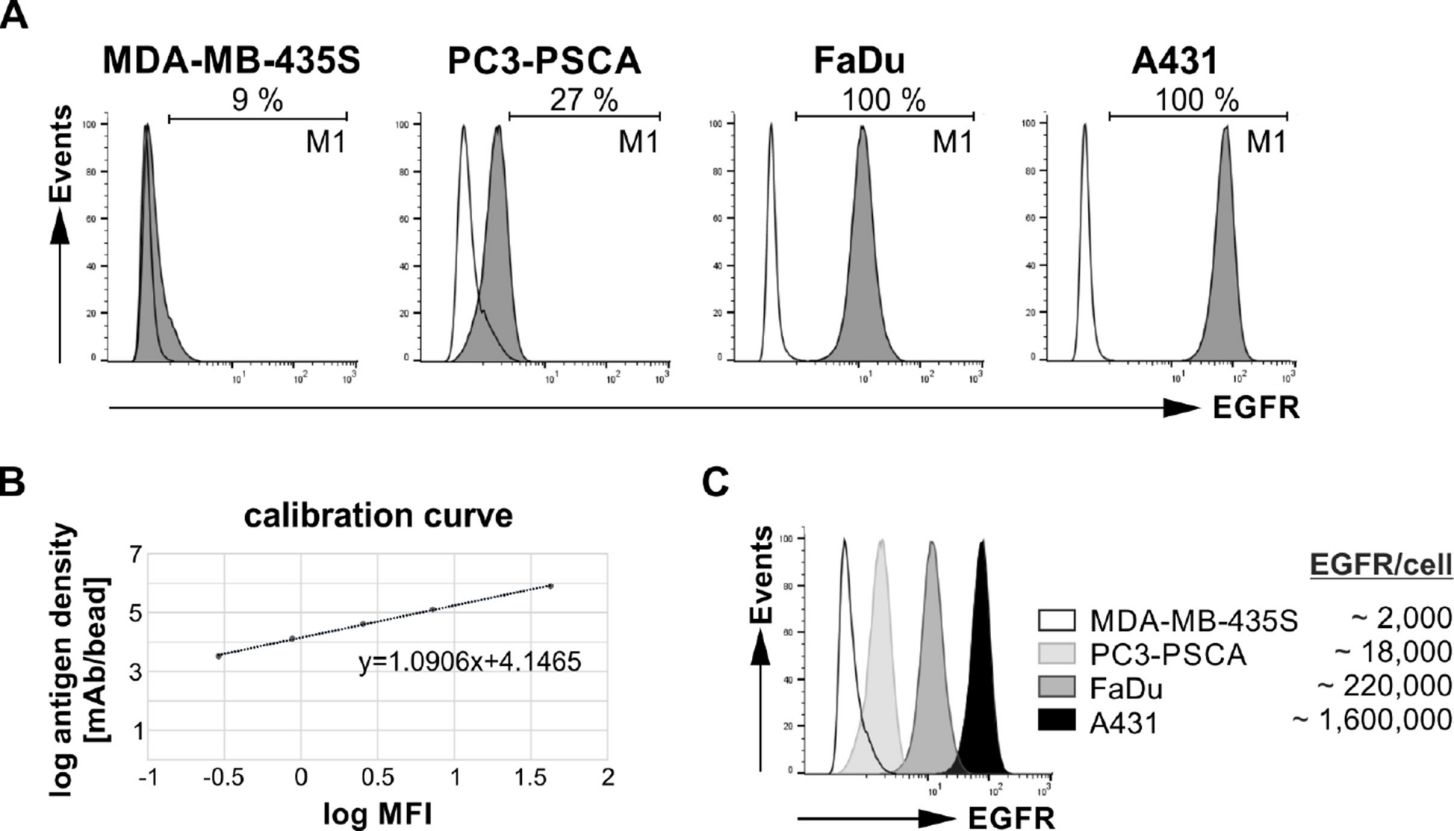
EGFR expression on different human tumor cell lines Analysis of cell surface EGFR expression level was performed by flow cytometry using fluorescence-based QIFIKIT^®^. For antigen detection an α-EGFR IgG1 mAb and a FITC-conjugated α-mouse IgG mAb were applied. (**A**) Histograms show staining of MDA-MB-435S, PC3-PSCA, FaDu or A431 cells (gray graphs) in comparison to the respective controls (transparent graphs). The corresponding percentage of EGFR-positive cells is displayed under the marker M1. (**B**) To calculate the EGFR antigen density a calibration curve was generated by using calibration beads. (**C**) Comparison of EGFR-binding on different tumor cell lines and the respective number of EGFR molecules per cell.

In a first step, we estimated EGFR surface expression levels of tumor cells by flow-cytometry on the surface of different tumor cell lines using a commercial anti-EGFR mAb and the fluorescence-based QIFIKIT^®^. Four cell lines were selected namely A431, MDA-MB-435S, FaDu, and PC3-PSCA cells. PC3-PSCA cells represent PC3 cells that were previously manipulated to overexpress the prostate stem cell antigen (PSCA) [24]. As summarized in Figure 3, A431 cells exhibit the highest (1.6 × 10^6^ EGFR/cell) and MDA-MB-435S cells the lowest number of EGFR molecules per cell (2 × 10^3^ EGFR/cell). FaDu cells show an intermediate (2.2 × 10^5^ EGFR/cell) and PC3-PSCA cells a low EGFR surface expression (1.8 × 10^4^ EGFR/cell).

**Figure 4:**
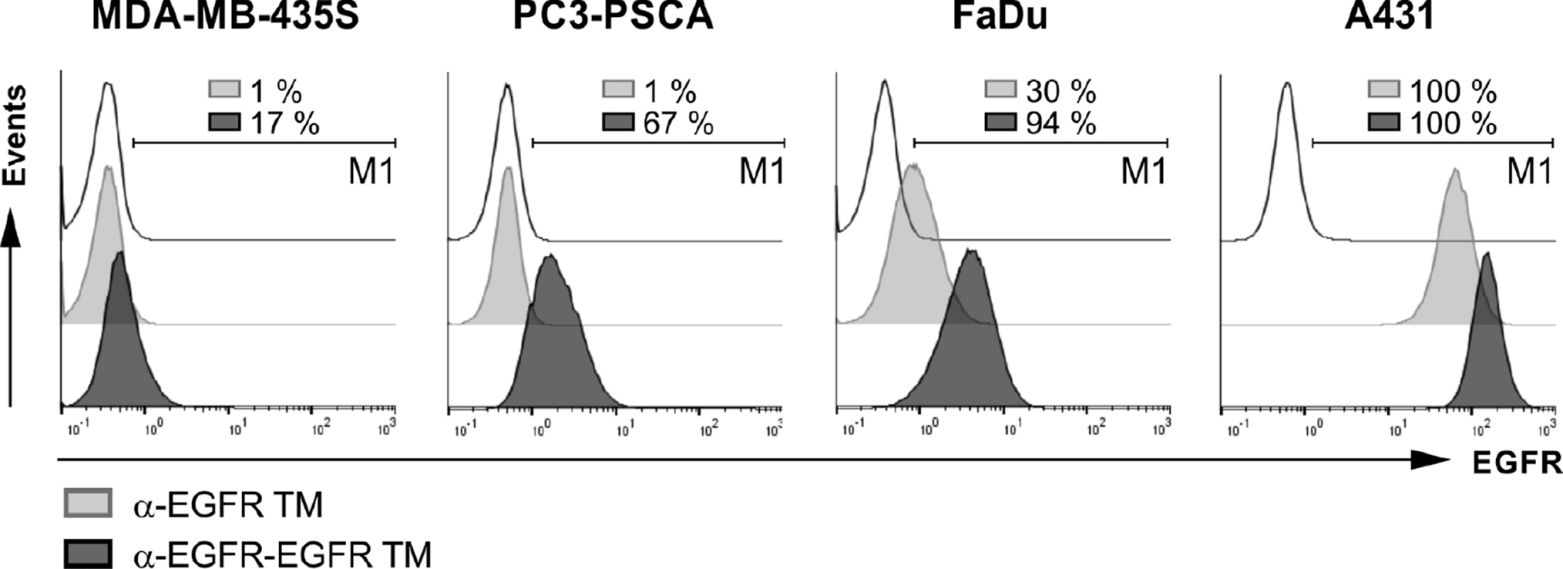
Binding of EGFR-specific TMs to antigen-expressing tumor cells To compare binding properties of the bivalent α-EGFR-EGFR TM with the monovalent α-EGFR TM, MDA-MB-435S, PC3-PSCA, FaDu and A431 cells were stained with 20 ng/ml of the respective construct. Specific binding was detected with an α-E5B9 mAb and a Pacific Blue™-conjugated α-mouse-IgG (Fcγ) mAb. Histograms show different tumor cell lines stained with the bivalent TM (dark gray graphs) or the monovalent TM (light gray graphs) in comparison to the respective controls (transparent graphs). In order to estimate the percentage of cells positively stained with the TMs, the marker M1 was set relative to the corresponding negative control. Respective numbers are given in each histogram. One representative experiment of three is shown.

Binding of both the monovalent α-EGFR TM and the bivalent α-EGFR-EGFR TM to these EGFR^+^ cell lines was compared by flow cytometry analysis (Figure 4). Binding was detected via the mAb directed against the UniCAR epitope. As shown in Figure 4, the bivalent α-EGFR-EGFR TM is able to bind to all tested tumor cell lines although the percentage of bound target cells varies in dependence on the EGFR expression level. In contrast, tumor cell binding of the monovalent α-EGFR TM could only be verified for cell lines with high or intermediate EGFR expression namely A431 cells and FaDu cells.

These data also show that the UniCAR epitope present in both the mono- and bivalent TM is accessible for interactions with an Ab domain directed against this epitope even after binding of the respective TM to EGFR. This is an important prerequisite for the interaction of surface-bound TMs with UniCAR-equipped T cells.

**Figure 5:**
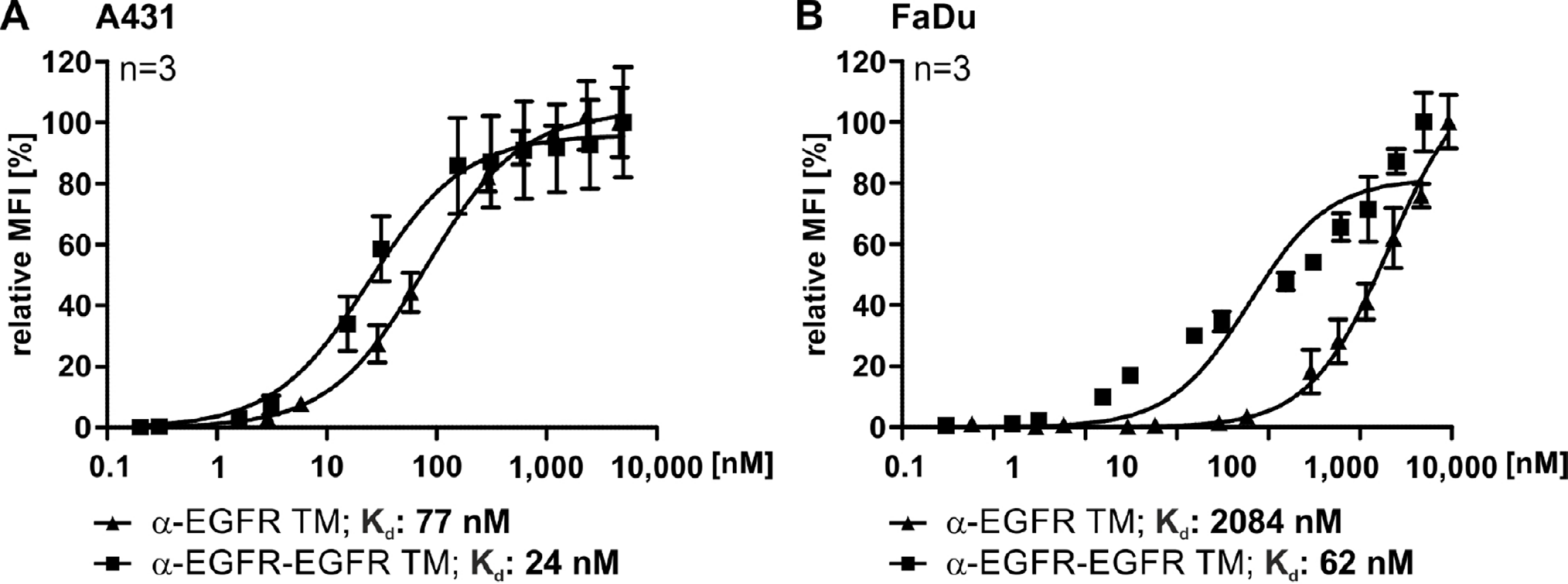
Comparison of the affinities of the mono- and bivalent EGFR-specific TMs For estimation of the respective K_d_ value increasing amounts of monovalent α-EGFR TM (triangle) or bivalent α-EGFR-EGFR TM (rectangle) were used to stain EGFR-positive (**A**) A431 or (**B**) FaDu cells. Detection of the specific binding was performed via the E5B9-tag with an α-E5B9 mAb and a Pacific Blue^™^-conjugated α-mouse-IgG (Fcγ) mAb. The resulting binding curves were used to calculate the K_d_ values. Average relative MFI and SD of three independent experiments are shown.

Finally, we determined the K_d_ values for binding of both the α-EGFR-EGFR TM and the α-EGFR TM to A431 or FaDu cells (Figure 5). In case of A431 cells, we estimated a K_d_ value of 24 nM for the α-EGFR-EGFR TM and a K_d_ value of 77 nM for the α-EGFR TM (Figure 5A). The superiority of the bivalent construct could be further confirmed by using FaDu cells (Figure 5B). With a K_d_ value of 62 nM the avidity of the bivalent TM increased 34-fold in comparison to the monovalent α-EGFR TM showing a K_d_ value of 2084 nM.

### Effect of the novel bivalent α-EGFR-EGFR TM on EGFR signaling

**Figure 6:**
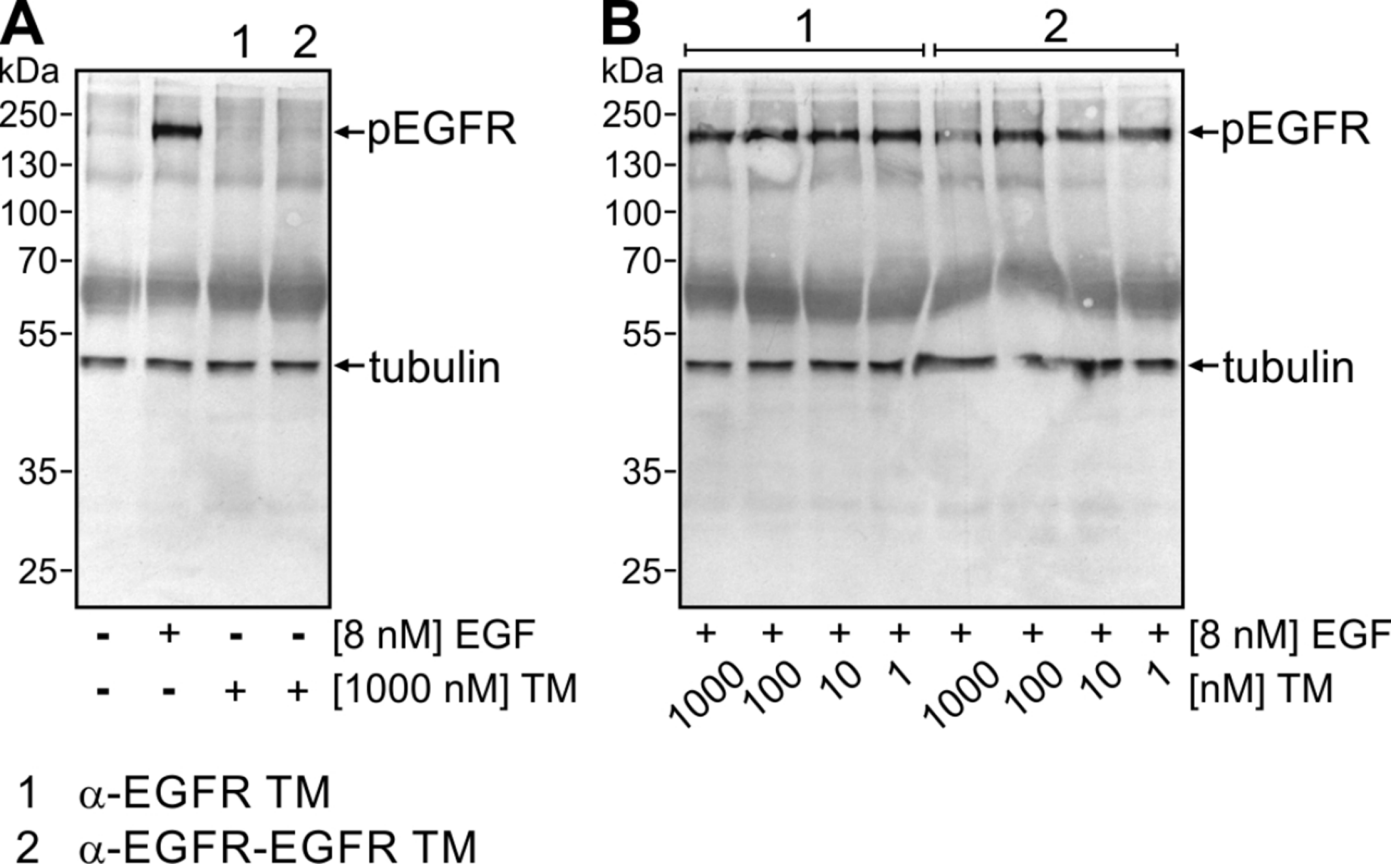
Influence of the mono- and bivalent EGFR-specific TMs on EGFR signaling (**A**) Serum-starved A431 tumor cells were incubated at 37° C either with no ligand, 8 nM EGF, 1000 nM α-EGFR TM (lane 1) or 1000 nM α-EGFR-EGFR TM (lane 2). After 15 min, cells were harvested and total cell lysates were separated by SDS-PAGE and subsequently transferred onto a nitrocellulose membrane. Afterwards, membranes were stained with mAbs against phosphorylated EGFR (pEGFR) and β-tubulin as loading control. (**B**) Mixtures of EGF (8 nM) and increasing amounts of the respective TM between 1 nM and 1000 nM were added to serum-starved A431 cells. Immunochemical detection of pEGFR was performed as described in (A).

Next, we analyzed whether or not the binding of the EGFR-specific TMs has an influence on EGFR signaling. For this purpose, phosphorylation status of tyrosine 1068 of the EGFR was investigated. This phosphorylation site is the starting point of the Ras signaling pathway and docking site for Grb2 [37]. To look for potential agonistic effects of both the α-EGFR-EGFR TM or the α-EGFR TM, A431 tumor cells were incubated for 15 min at 37° C in the absence or presence of EGF or the respective TM instead (Figure 6A). As expected EGF induced phosphorylation of EGFR. In contrast, no specific EGFR phosphorylation was detected upon binding of the TMs. According to these data, the Nb-based constructs have no intrinsic activity under the tested circumstances. Furthermore, we analyzed the ability to block EGF-induced phosphorylation. For this purpose, A431 cells were incubated with a mixture of EGF (8 nM) and increasing amounts of the respective TM (1 nM to 1000 nM). As shown in Figure 6B, none of the tested concentrations of the monovalent α-EGFR TM blocked EGF effects. In general, results obtained with the bivalent α-EGFR-EGFR TM were similar though at the highest TM concentration a slight inhibition was detectable. However, such a high TM concentration would never be reached during treatment of a patient as shown below (see estimation of EC_50_ value (Figure 8) and discussion section). Hence, the TM binding to EGFR does neither cause nor interfere with EGF-mediated signaling at least under retargeting conditions.

**Figure 7:**
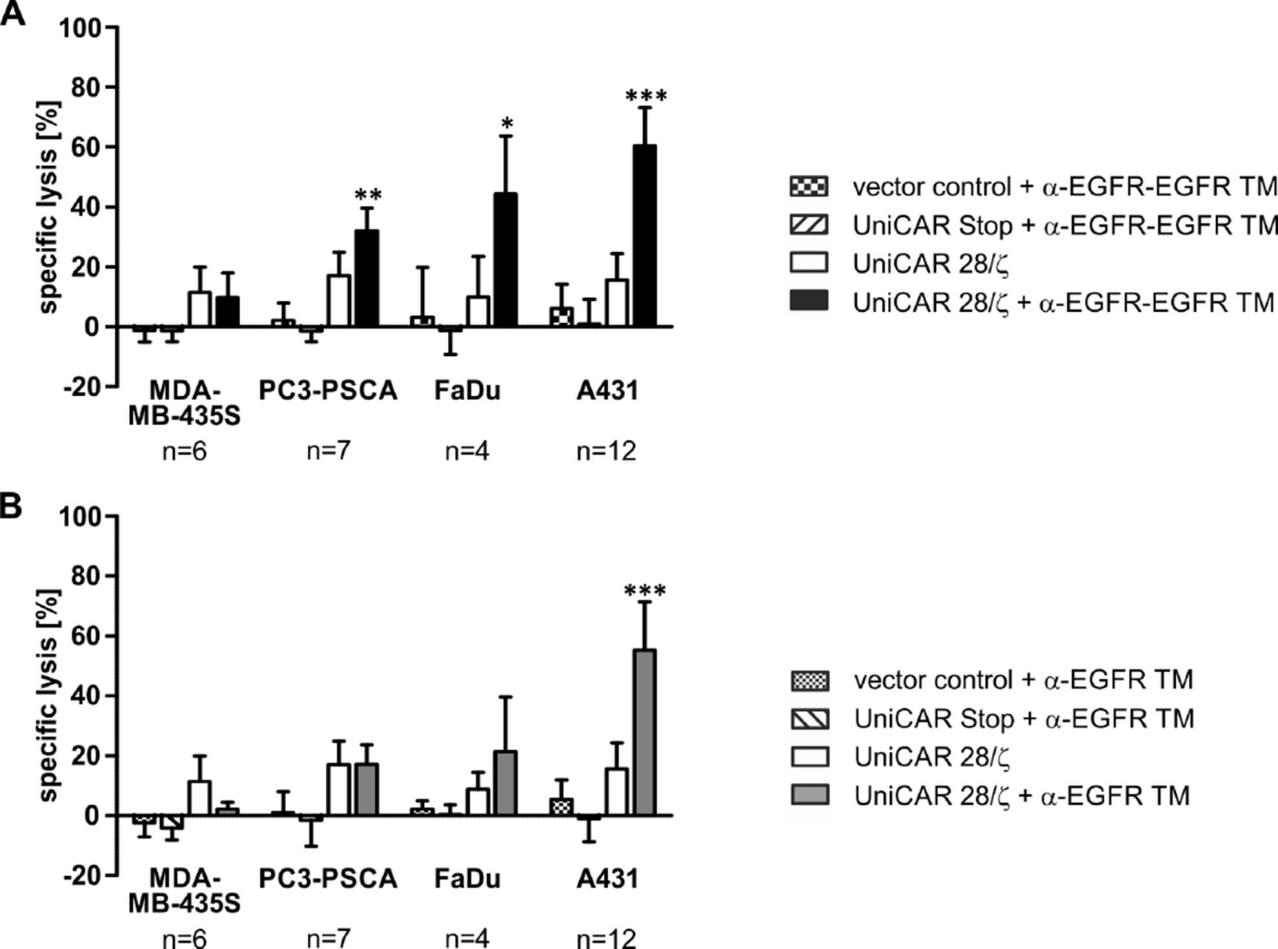
Retargeting of UniCAR-armed T cells against EGFR-positive tumor cell lines via EGFR-specific TMs Killing properties were analyzed by standard chromium release assays. Therefore, T cells engrafted with the EGFP-expressing vector control, the UniCAR Stop construct lacking intracellular signaling domains or the UniCAR 28/ζ-signaling construct were incubated with ^51^Cr-loaded MDA-MB-435S, PC3-PSCA, FaDu or A431 cells. The co-cultivation of T cells and tumor cells was performed at an effector to target cell ratio of 5:1 in the presence or absence of 50 nM (**A**) α-EGFR-EGFR TM or (**B**) α-EGFR TM for 48 h. Average specific lysis and SD for six (MDA-MB-435S), seven (PC3-PSCA), four (FaDu) or twelve (A431) independent T cell donors are shown (^*^p < 0.05, ^**^p < 0.01, ^***^p < 0.001; with respect to controls: vector control or UniCAR Stop + TM and UniCAR 28/ζ; one-way ANOVA with Bonferroni multiple-comparison test).

**Figure 8:**
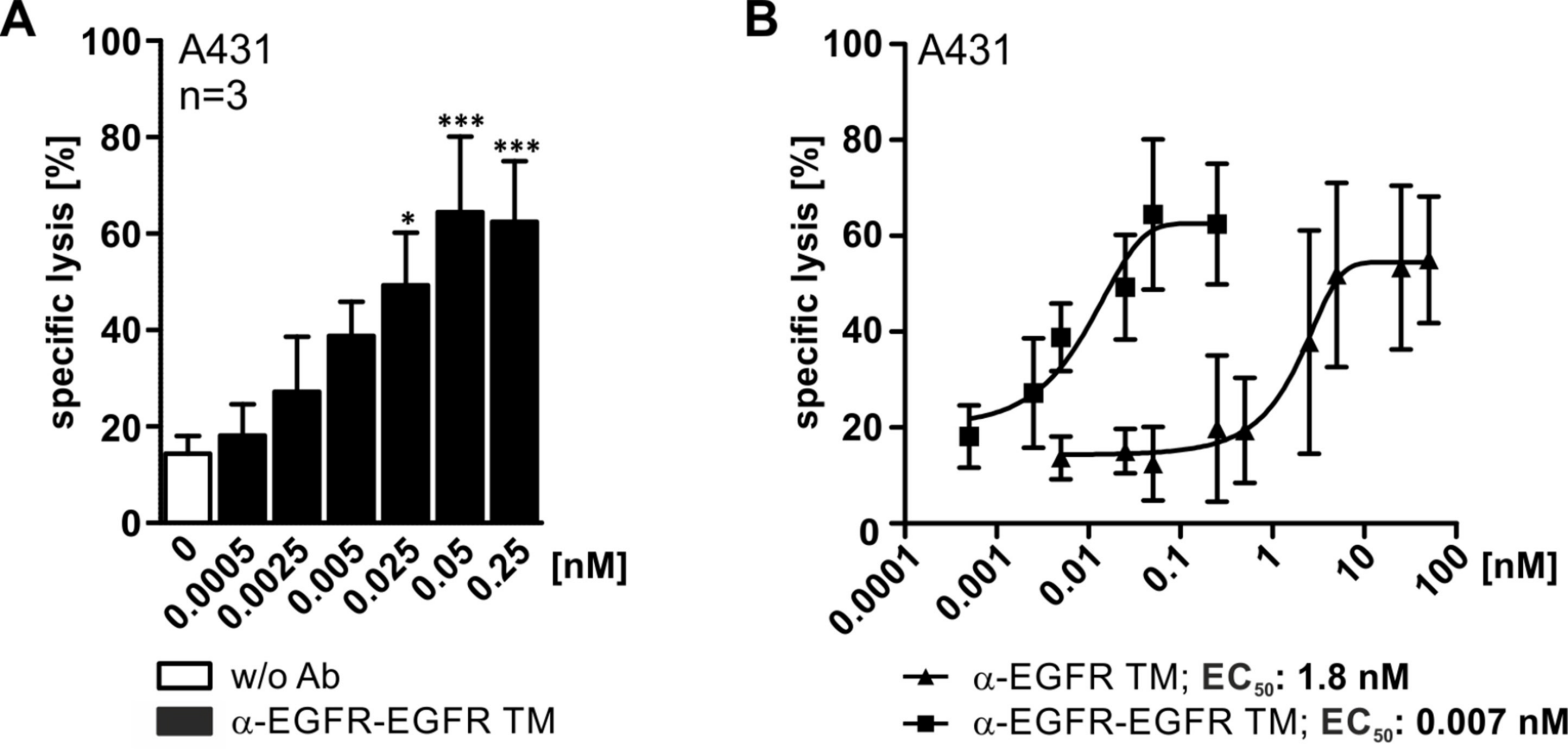
Comparison of working concentrations for the mono- and bivalent EGFR-specific TMs To estimate the TM-dependent killing efficiency of UniCAR T cells standard chromium release assays were performed. UniCAR 28/ζ-armed T cells were cultivated with ^51^Cr-labeled A431 cells in an effector to target cell ratio of 5:1 with increasing amounts of EGFR-specific TM for 48 h. (**A**) Shown data represent mean specific lysis and SD for three independent T cell donors incubated with the bivalent α-EGFR-EGFR TM (^*^p < 0.05, ^***^p < 0.001; with respect to control: UniCAR 28/ζ w/o Ab; one-way ANOVA with Bonferroni multiple-comparison test). (**B**) Based on the specific lysis of chromium release assays the EC_50_ values were calculated. The graph displays the comparison of the calculated killing curves for the monovalent and the bivalent EGFR-specific TM. Mean specific lysis and SD for five (monovalent TM) or three (bivalent TM) independent T cell donors are shown.

### Redirection of UniCAR-armed T cells via the novel bivalent TM enhances lysis of EGFR-expressing tumor cells

In order to analyze whether the increased avidity of the α-EGFR-EGFR TM influences its ability to redirect UniCAR-engrafted T cells for an efficient killing of different EGFR^+^ cancer cells, chromium-based killing assays were performed. Therefore, human T cells were transduced with a lentiviral vector encoding the UniCAR 28/ζ signaling-construct. Genetically modified T cells expressing solely EGFP (vector control) or the UniCAR without intracellular signaling domains (UniCAR Stop) served as negative controls. Tumor cell lysis was determined by measuring chromium release after 48 h of co-cultivation (Figures 7 and 8). Analysis was carried out in comparison to the monovalent α-EGFR TM.

As shown in Figure 7A, UniCAR 28/ζ-modified T cells are able to efficiently eradicate tumor cells in the presence of the α-EGFR-EGFR TM. In line with the TM-binding data (Figure 4), tumor cell lysis correlates with the antigen density on the respective target cells. Highest lysis rate was observed for A431 cells, intermediate to low lysis rate for FaDu and PC3-PSCA cells, respectively, while MDA-MB-435S cells were not eliminated (Figure 7A). In contrast to the bivalent α-EGFR-EGFR TM, the monovalent α-EGFR TM is less efficient and mediates solely a significant eradication of cancer cells with high EGFR density (Figure 7B). No considerable tumor cell killing by UniCAR 28/ζ-equipped T cells was measured in the absence of any TM. Additionally, UniCAR Stop- and vector control-transduced T cells were not able to lyse target cells.

In a next step, ^51^Cr-labeled A431 tumor cells and UniCAR 28/ζ-armed T cells were incubated with different concentrations of the TMs ranging between 0 and 50 nM (Figure 8). The obtained data clearly underline that the bivalent α-EGFR-EGFR TM is superior to the monovalent α-EGFR TM regarding functionality. UniCAR T cell-mediated lysis of A431 cells already reached a maximum efficiency at concentrations of 0.05 nM for the bivalent α-EGFR-EGFR TM, while 100-fold higher concentrations of the monovalent α-EGFR TM were required. This is in line with the EC_50_ value calculated for the bivalent α-EGFR-EGFR TM that is 250 times lower (0.007 nM) than that of the monovalent α-EGFR TM (1.8 nM). As also seen for previously described TMs, tumor cell lysis varied in a wide range between the different T cell donors.

### Cytokine release of cross-linked UniCAR-armed T cells

**Figure 9:**
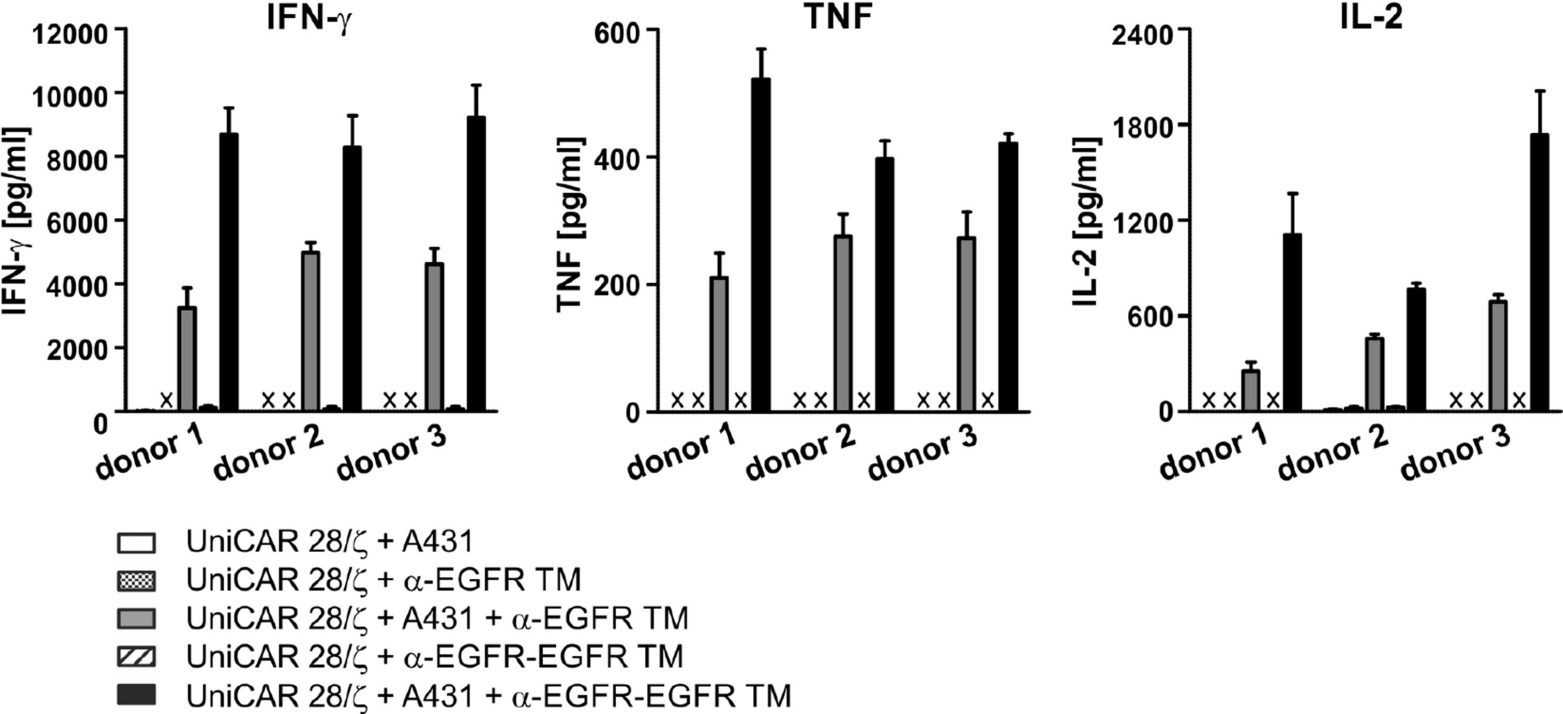
ELISA analysis of cytokine release from EGFR-redirected UniCAR-endowed T cells UniCAR 28/ζ-armed T cells were cultivated with A431 tumor cells in the presence or absence of 50 nM α-EGFR TM or α-EGFR-EGFR TM at an effector to target cell ratio of 5:1. As controls UniCAR 28/ζ-modified T cells were incubated with the respective TM in the absence of EGFR-positive target cells. After 48 h, concentrations of the cytokines IFN-γ, TNF and IL-2 were estimated in cell culture supernatants by ELISA. The average cytokine concentration and SD from experiments for three independent donors are shown (x, not detectable).

Based on previous studies, including for the monovalent α-EGFR TM using a multiplex assay (the MACSPlex Cytokine 12 Kit) we know that the major cytokines released from UniCAR-armed T cells are GM-CSF, IFN-γ, IL-2, and TNF. Other cytokines including IL-4, IL-5, IL-6, IL-9, IL-10, IL-12, IL-17A could not be detected at a significant concentration [23–25]. Therefore, in this study we focused on the cytokines IFN-γ, TNF and IL-2. To estimate secreted concentrations, UniCAR 28/ζ-equipped T cells were co-cultivated with A431 tumor cells (expressing high levels of EGFR) in the absence or presence of either the mono- or bivalent EGFR-specific TM. Additionally, effector T cells were incubated solely with the TMs in the absence of A431 cells to exclude unspecific TM effects. After 48 h of cultivation, cell culture supernatants were analyzed by ELISA for release of IFN-γ, TNF and IL-2 (Figure 9). In general, cytokines were only detected after incubation of UniCAR 28/ζ-armed T cells and tumor cells in the presence of TMs. Thus, cytokine release is strictly dependent on the TM-mediated cross-linkage between UniCAR 28/ζ T cells and tumor cells. The bivalent TM triggers a higher release of pro-inflammatory cytokines IFN-γ and TNF as well as growth-promoting cytokine IL-2 in comparison to the monovalent TM. Besides, IFN-γ represented the most prominent cytokine secreted by EGFR-redirected UniCAR-armed T cells.

### Superior *in vivo* anti-tumor reactivity of the bivalent TM

**Figure 10:**
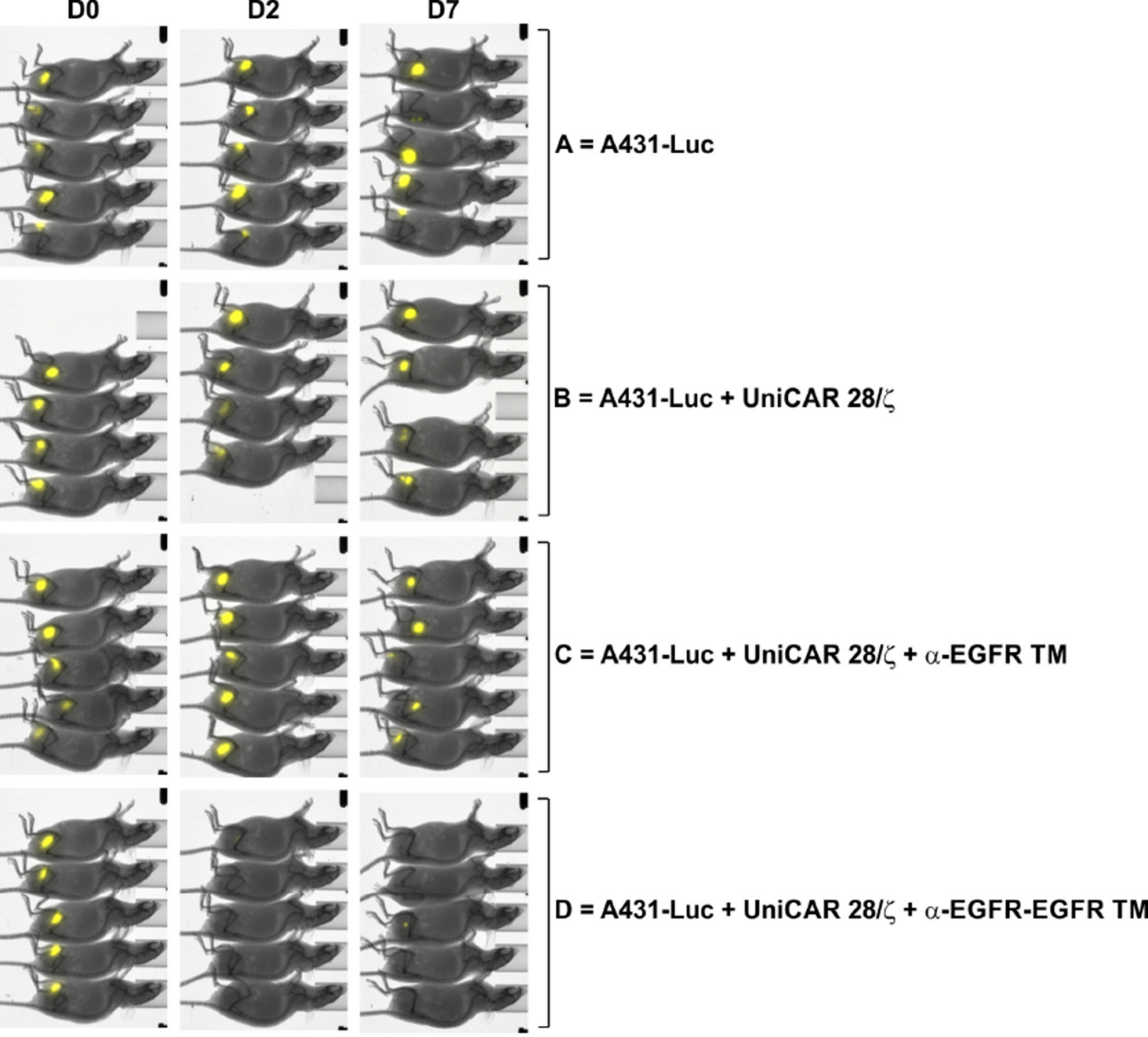
UniCAR system-based retargeting of EGFR-positive tumor cells in experimental mice Comparison of monovalent versus bivalent TM. For each mouse of the treatment group mixtures of 1.5 x 10^6^ A431-Luc cells, 1.5 x 10^6^ UniCAR 28/ζ-armed T cells and 600 pmol of the monovalent α-EGFR TM (group C) or bivalent α-EGFR-EGFR TM (group D) were prepared. As untreated controls served A431-Luc cells alone (group A) or A431-Luc cells mixed with UniCAR 28/ζ-equipped T cells (group B). The respective mixtures were subcutaneously injected into the right flank of female NMRI^nu/nu^ mice resulting in four groups of animals. At day 0 (D0), day 2 (D2) and day 7 (D7) bioluminescence imaging of anesthetized mice was performed 10 min after i.p. injection of 200 µl luciferin (15 µg/µl).

By using an established xenograft mouse tumor model, the functionality of the monovalent α-EGFR TM and the bivalent α-EGFR-EGFR TM was finally compared *in vivo*. As described recently, A431 cells genetically modified to express firefly luciferase (termed A431-Luc) served as target cells [23]. Under the chosen experimental conditions, UniCAR-modified T cells armed with the monovalent α-EGFR TM were able to completely eliminate the injected tumor cells [23]. In order to challenge the question whether or not the improved *in vitro* killing efficacy of the bivalent α-EGFR-EGFR TM turns also into an improved anti-tumor functionality *in vivo*, we reduced the amount of TMs ten times (down to 600 pmol per mouse) compared to the previously described experiment. Under these circumstances, we expected to see little if any effect by administering the monovalent α-EGFR TM, while the bivalent α-EGFR-EGFR TM should still be functional. For this experiment, 1.5 × 10^6^ A431-Luc cells were mixed with UniCAR 28/ζ-armed T cells at an effector-to-target cell ratio of 1:1 and the respective TM. A431-Luc cells alone or mixed with UniCAR 28/ζ T cells without any TM served as negative controls. The respective mixtures were subcutaneously injected into female NMRI^nu/nu^ mice resulting in four groups of animals (Figure 10). By performing bioluminescence imaging, the tumor growth of luciferase-expressing cells was monitored. All mice were analyzed in parallel at day 0 (Figure 10, D0), day 2 (Figure 10, D2) and day 7 (Figure 10, D7). As shown in Figure 10, A431 Luc cells were visible in all experimental mice including the treatment groups directly after s.c. injection of the respective TM/cell mixtures. Seven days later, tumor cells could no longer be detected in the treatment group receiving the bivalent α-EGFR-EGFR TM. In contrast, luciferase activity was still visible in both control animals and experimental mice treated with the monovalent α-EGFR TM. These data indicate that under the applied experimental conditions only the bivalent α-EGFR-EGFR TM was able to successfully redirect UniCAR-armed T cells against EGFR-positive tumor cells *in vivo* thus underlining the superiority of the bivalent α-EGFR-EGFR TM.

### Biodistribution of the bivalent α-EGFR-EGFR TM in tumor bearing mice

For analysis of the biodistribution and estimation of the pharmacokinetic behavior of the TM both the monovalent α-EGFR TM and the bivalent α-EGFR-EGFR TM were radiolabeled. Prior to radiolabeling, we had to remove the contaminating HMWs [25]. The resulting purity of purified α-EGFR-EGFR TM was better than 95% as estimated by SDS-PAGE (Supplementary Figure 1A) and HPLC (data not shown). Purified TMs were conjugated with the chelator NODAGA [23, 25] (see Materials and Methods). After conjugation the modified α-EGFR-EGFR-NODAGA TM was functionally compared with the unmodified α-EGFR-EGFR TM. As shown in Supplementary Figure 1B, the conjugation of NODAGA did not impair the functionality of the α-EGFR-EGFR TM. NODAGA can be used for chelating of ^64^Cu. The resulting radiolabeled bivalent [^64^Cu]Cu-α-EGFR-EGFR-NODAGA TM and monovalent [^64^Cu]Cu-α-EGFR-NODAGA TM had a molar activity larger than 24 GBq/µmol and was of high radiochemical purity (>95%).

**Figure 11:**
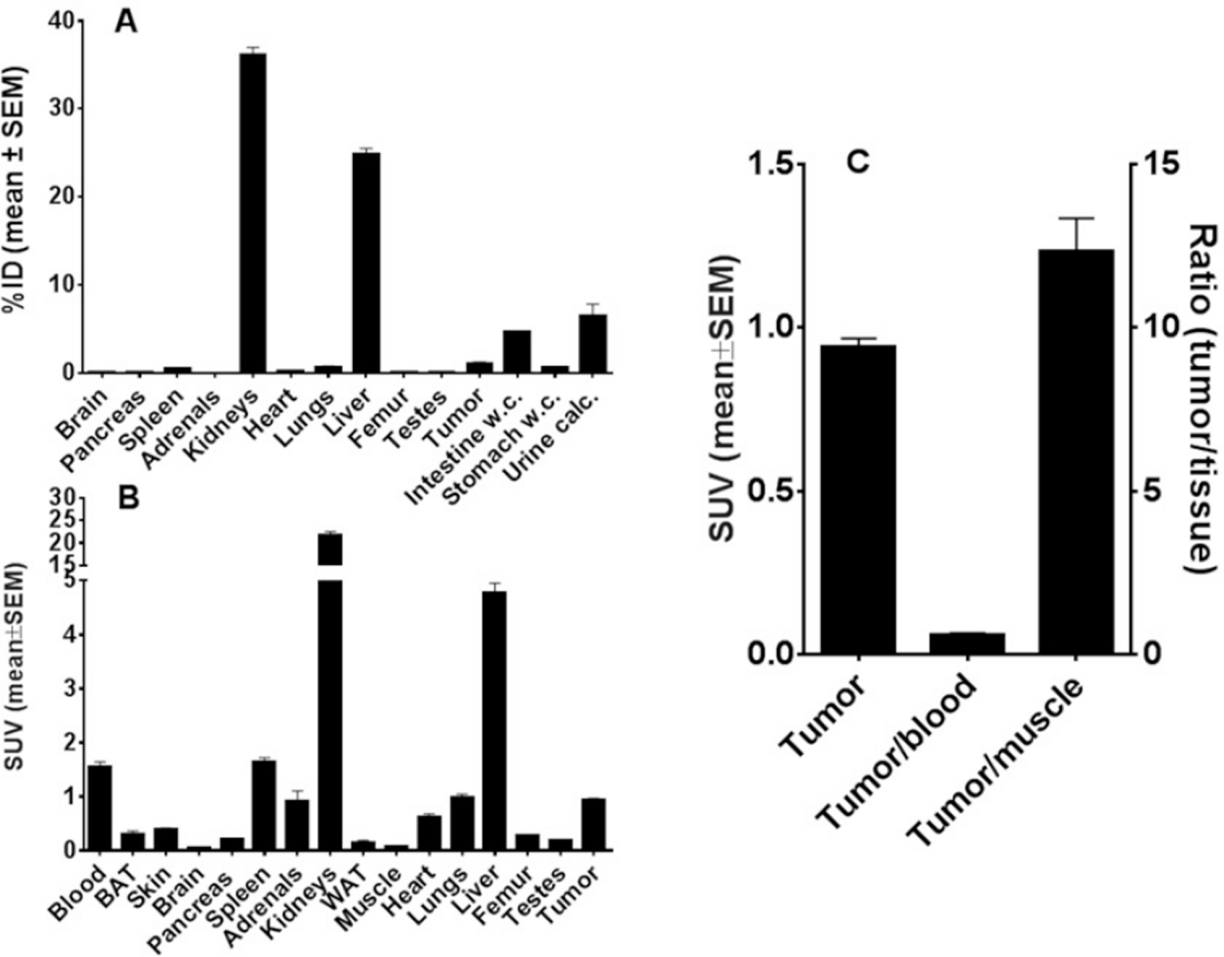
Biodistribution of ^64^Cu-radiolabeled α-EGFR-EGFR TM After conjugation of the α-EGFR-EGFR TM with NODAGA the resulting α-EGFR-EGFR-NODAGA TM was radiolabeled with ^64^Cu. The biodistribution of the [^64^Cu]Cu-α-EGFR-EGFR-NODAGA TM complex is shown in (**A, B**). (**A**) The biodistribution is given as percentage of the total activity of the injected dose (%ID) and (**B**) the activity concentration (SUV) based on four A431-Luc tumor bearing mice. (**C**) Target to background ratios including tumor-to-muscle-, and tumor-to-blood ratios. The data were collected 2 h after injection of the [^64^Cu]Cu-α-EGFR-EGFR-NODAGA TM.

The biodistribution of the [^64^Cu]Cu-α-EGFR-EGFR-NODAGA TM is summarized in Figure 11. Diagrams show activity amounts in the whole organs either as percentage of the total activity of the injected dose (%ID) (Figure 11A) or the activity concentration (SUV) for a total of four A431-Luc tumor bearing mice (Figure 11B) 2 h after injection. Target to background ratios including tumor-to-muscle-, and tumor-to-blood ratios are shown in Figure 11C. Data reveal that the bivalent TM is eliminated via glomerular filtration into the urine as well as via the hepatobiliary system. This is in contrast to the monovalent TM, which is mainly eliminated via the kidneys [23].

**Figure 12:**
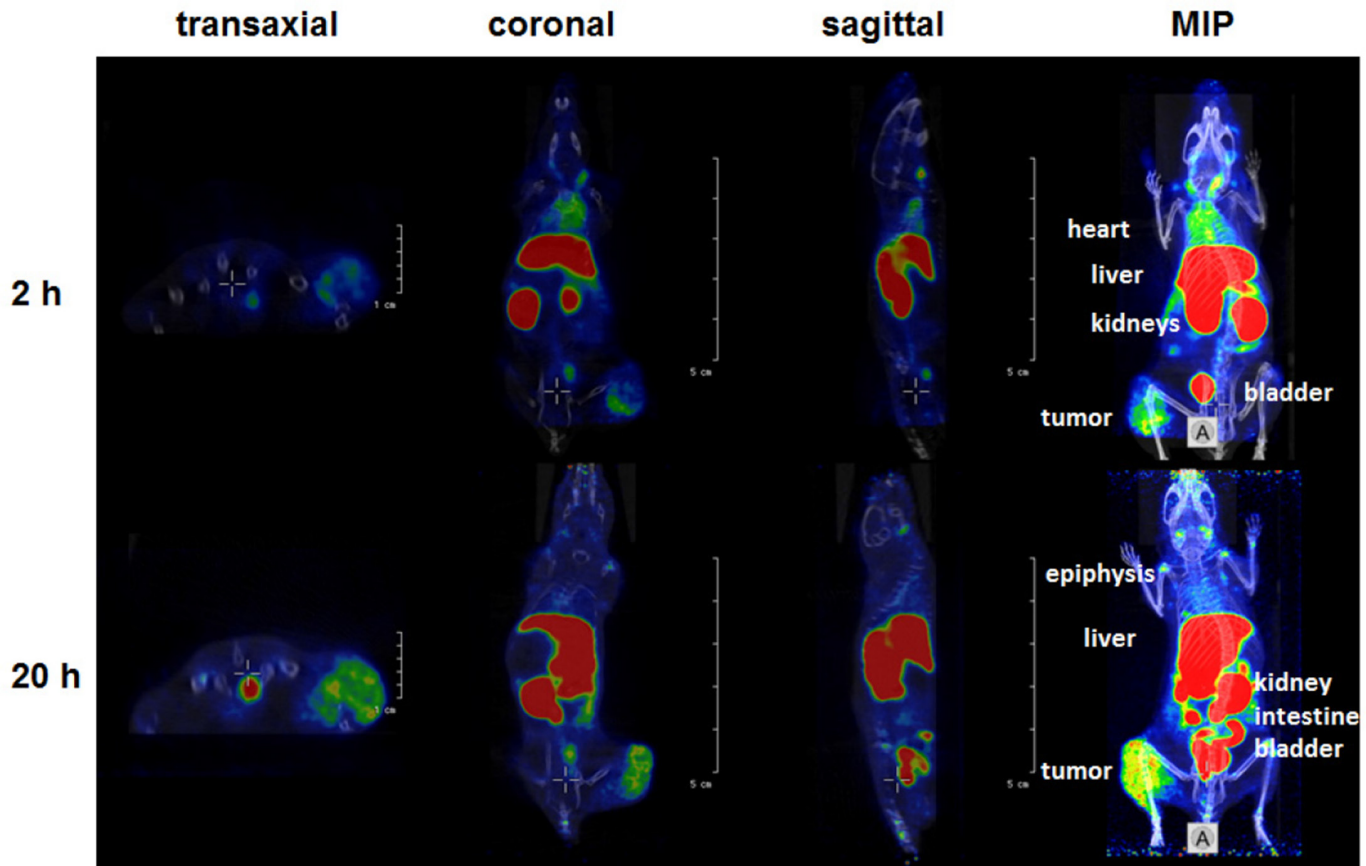
Small animal PET/CT Orthogonal sections (transaxial, coronal, sagittal) scaled to visualize the tumor or maximum intensity projections (MIP) of a selected A431-Luc tumor bearing mouse at 2 h or 20 h after single intravenous injection of the [^64^Cu]Cu-α-EGFR-EGFR-NODAGA TM complex. Note: The [^64^Cu]Cu-α-EGFR-EGFR-NODAGA TM complex further accumulates at the tumor site between 2 h and 20 h after injection (see also Figure 13B).

As shown by small animal PET/CT the [^64^Cu]Cu-α-EGFR-EGFR-NODAGA TM enriched at the tumor site already after 2 h (Figure 12, upper panel). However, the maximal tumor activity concentration and maximal contrast was not reached at this time point. A portion of the tracer [^64^Cu]Cu-α-EGFR-EGFR–NODAGA TM can still easily be detected in the blood and heart of the analyzed mouse, which is consistent with the high blood activity concentration measured in the biodistribution (Figure 11). Thus, the tracer can further enrich at the tumor site during the following 18 h (Figure 12, lower panel).

**Figure 13:**
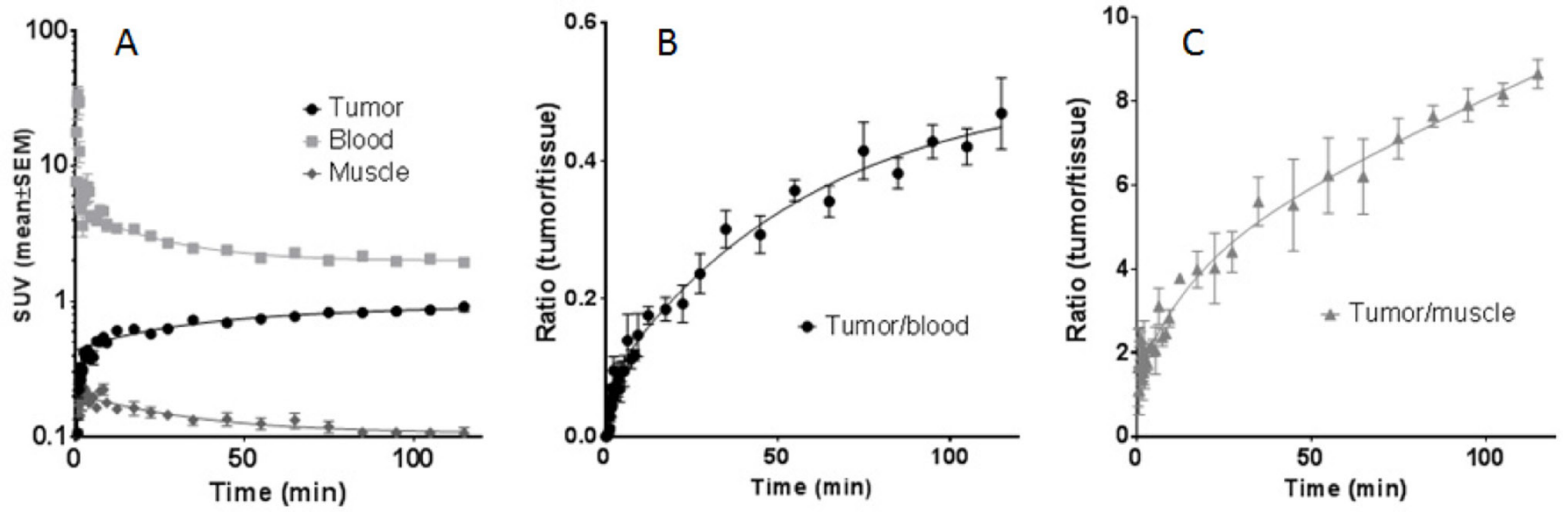
Time-activity curves (TAC) of regions of interest (ROI) The TAC curves are derived from PET studies of four A431-Luc tumor bearing mice after injection of the [^64^Cu]Cu-α-EGFR-EGFR-NODAGA TM complex as indicated. (**A**) TAC representing primarily the tumor, blood, and muscle activity concentration (SUV_mean_). (**B**), (**C**) TAC of the tumor ratios to blood and muscle, respectively, supporting the increasing image contrast even beyond 2 h.

**Figure 14:**
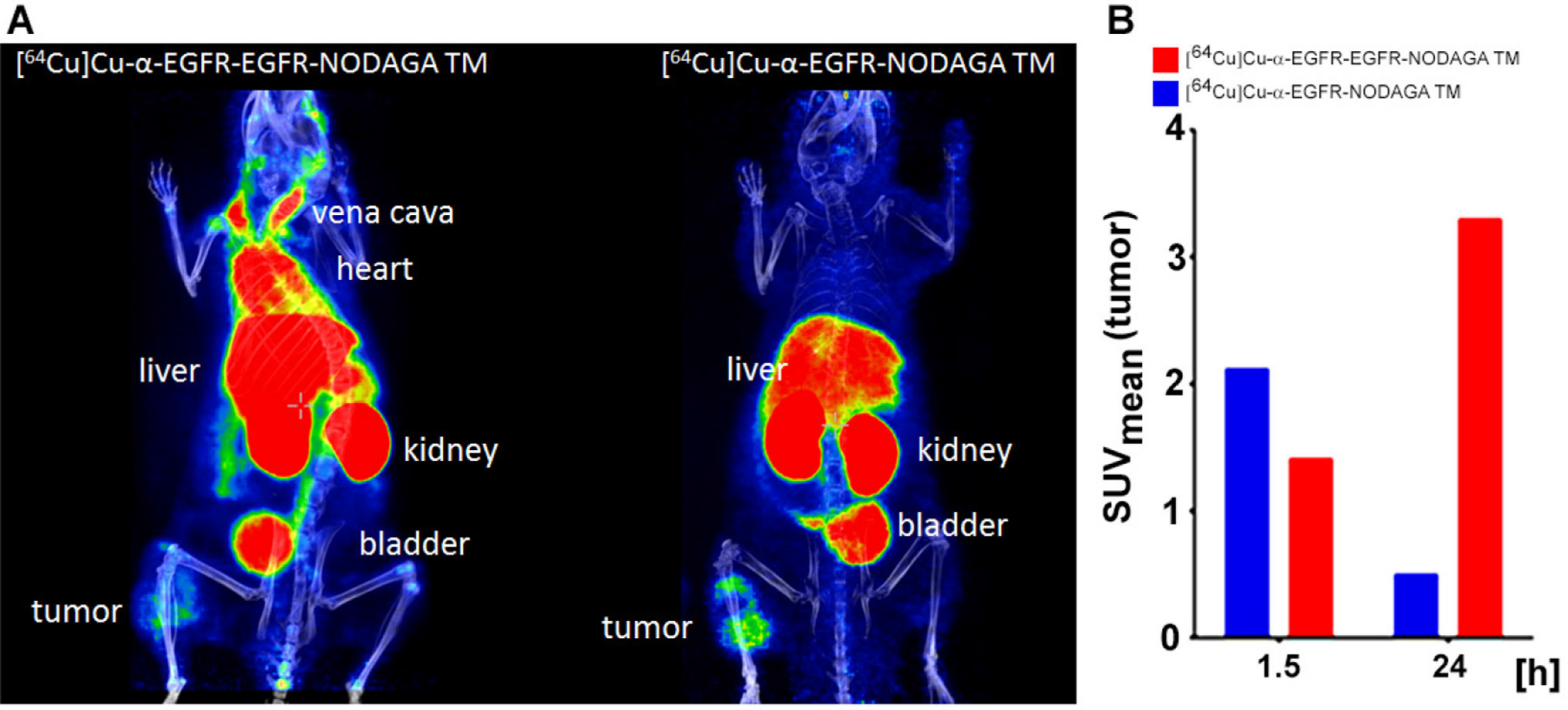
Small animal PET analysis of selected A431-Luc tumor bearing mice after single intravenous injection of the [^64^Cu]Cu-α-EGFR-EGFR-NODAGA TM- or the [^64^Cu]Cu-α-EGFR-NODAGA TM complex (**A**) PET/CT scan scaled to maximum intensity projection at 2 h after injection of the [^64^Cu]Cu-α-EGFR-EGFR-NODAGA TM complex (left panel) or the [^64^Cu]Cu-α-EGFR-NODAGA TM complex (right panel). Note: At this time point the [^64^Cu]Cu-α-EGFR-EGFR-NODAGA TM complex can still be detected in the blood (heart) (left panel). In contrast, the [^64^Cu]Cu-α-EGFR-NODAGA TM complex can hardly be detected in the blood (right panel). (**B**) The [^64^Cu]Cu-α-EGFR-EGFR-NODAGA TM complex has not reached the maximum concentration in the tumor at 1.5 h after injection. The concentration can further increase in the tumor during the following 24 h (red bars). In contrast, the [^64^Cu]Cu-α-EGFR-NODAGA TM complex has already reached the maximum concentration in the tumor during the first 1.5 h after injection and thus the concentration in the tumor decreases during the following 24 h (blue bars).

In order to support this pharmacokinetic behavior of the bivalent tracer [^64^Cu]Cu-α-EGFR-EGFR–NODAGA TM, time-activity curves (TAC) of regions of interest (ROI) derived from dynamic PET studies of four analyzed A431-Luc tumor bearing mice were estimated. The midframe data points were calculated over a time range of 2 h p.i. of the [^64^Cu]Cu-α-EGFR-EGFR-NODAGA TM (Figure 13). TAC representing primarily the tumor, blood, and muscle activity concentration estimated as SUV_mean_ are shown in Figure 13A. The respective curve of the blood indicates a rapid elimination of the bivalent TM with a serum half-life of only 9.3 min. Additionally, the TAC of the tumor shows that the TM has a clearance half-life of 5.2 h. TACs of the tumor-to-blood and tumor-to-muscle ratios, are presented in Figure 13B and Figure 13C, respectively. In agreement with the data presented in Figure 12, these results support the interpretation that the maximal image contrast is not reached 2 h p.i.. Altogether these data indicate that the pharmacokinetic behavior of the bivalent [^64^Cu]Cu-α-EGFR-EGFR-NODAGA TM differs from the previously described [23] monovalent [^64^Cu]Cu-α-EGFR-NODAGA TM as also supported by the side by side comparison of both radiolabeled TMs (Figure 14A). As mentioned above (and also seen in Figure 14A, left panel), 2 h p.i. the bivalent [^64^Cu]Cu-α-EGFR-EGFR-NODAGA TM is still detectable in the heart/blood stream of the experimental mouse. In contrast, the monovalent α-EGFR tracer is almost completely eliminated from the heart/blood stream at this time (Figure 14A, right panel). Therefore, the monovalent [^64^Cu]Cu-α-EGFR-NODAGA TM cannot further enrich at the tumor site but already decreases between 1.5 h and 24 h p.i. (Figure 14B, blue bars). In contrast, the bivalent [^64^Cu]Cu-α-EGFR-EGFR-NODAGA TM has not reached its maximum concentration 1.5 h p.i. and can further enrich at the tumor site during the following 22.5 h (Figure 14B, red bars, see also Figure 12).

## DISCUSSION

The key to success in development of novel promising immunotherapeutic approaches is based on two factors: (I) high effectiveness at (II) lowest possible side effects. Particularly CAR-armed T cells represent attractive candidates for highly effective cancer care. Although first CAR T cell therapies have finally entered the clinical routine for treatment of cancer patients [38, 39], there is still the need to further improve the safety of such “living drugs”. Most concerning side effects associated with CAR T cell therapy are CRS and uncontrollable “on-target, off-tumor” reactions against healthy tissues with low TAA expression [35]. As shown by a case report using Her2/neu-specific CAR T cells, these adverse reactions can even be fatal [40]. Considering the ubiquitous expression of the HER family members, targeting of EGFR requires a sophisticated safety management and has to go beyond the currently available pharmacological immunosuppression [35]. Keeping the long-term persistence of CAR-engrafted T cells in mind [41, 42], EGFR-targeted therapy requires the shutdown of adoptively transferred immune effector cells to avoid permanent destruction of healthy tissues. Thus, we came up with the idea to manage the safety issue by separating the functional domains of CAR constructs and developed a novel platform technology termed UniCAR [22–27]. This modular tumor targeting strategy is based on T cells which are genetically modified to express the UniCAR and are per se inactive. Their anti-tumor reactivity can only be switched on and off in dependence of tumor-specific TMs recognized by the UniCAR. Hence, tumor killing mediated by UniCAR-modified T cells occurs in a TM dose-dependent manner allowing an easy reactivation at any time via renewed TM supply, e.g. in case of tumor relapse. Through titration of the TM unwanted treatment-related toxicities, including CRS and toxic effects against healthy tissues, can be easily managed.

So far, we have established a series of TMs for the UniCAR system including against CD33, CD123, CD19, PSCA, PSMA, and GD2 [22–27]. All these and other constructs (Bachmann, unpublished) were functional without any further optimization, although affinity of these TMs to the respective target cell varied in a wide range. Until now, we therefore assumed that the affinity between the UniCAR domain and its cognate epitope (E5B9) on the TM is most important for functionality, while the affinity between the surface TAA and the TM may be less relevant. We further expected that increasing the affinity towards the target site may even be counterproductive as the TM may stick to the surface of dead or apoptotic target cells and is therefore unavailable for binding to the next tumor cell.

However, this view was recently challenged when we analyzed the properties of a Nb-based monovalent α-EGFR TM that was expressed either in *E. coli* or in CHO cells [23]. Both EGFR-specific TM preparations were proven to be suitable for cancer immunotherapy and PET-imaging of tumors. In experimental mice, the TMs were rapidly eliminated from the circulation and from UniCAR-TM complexes, thus fulfilling all requirements for safety management by TM dosing. Yet for an unknown reason, the α-EGFR TM (pro) had a higher affinity and was more efficient than the α-EGFR TM expressed by eukaryotic cells. It remained, however, unclear whether or not the improved functionality of the TM expressed in prokaryotic cells was due to its higher affinity towards the target antigen. Nonetheless, these data demonstrate that the efficacy of the α-EGFR TM does not solely depend on its primary sequence and can be improved perhaps by changing the affinity towards the TAA.

To rule out effects caused by different expression systems and to obtain a TM with an enhanced affinity towards EGFR, we decided to construct a bivalent TM and expressed it with the same eukaryotic CHO cell system like the monovalent α-EGFR TM. Both EGFR-specific constructs are based on the camelid Nb clone 7C12. Since Roovers *et al.* reported (i) an inhibition of EGF-mediated EGFR phosphorylation in the presence of the EGFR-specific Nb 7D12 [43], and (ii) also mentioned a blockage of EGF binding by the Nb 7C12 used in our studies [43] we analyzed the capability of our α-EGFR TMs to mediate EGFR signaling and blocking of EGF binding to EGFR. Unfortunately, the data mentioned for the Nb 7C12 were not shown in detail making a direct comparison with our data difficult. Nonetheless, at least under our retargeting conditions we do not see either an intrinsic receptor activation or blockage of EGF-mediated EGFR phosphorylation. We only see a slight blocking effect at a concentration of the bivalent TM that occurs at around 10^5^ times the EC_100_ concentration requested for retargeting of UniCAR T cells. Consequently, such an unrealistic high TM concentration should never be reached under retargeting conditions in a patient and we therefore do not expect any interference of our TMs with EGFR signaling.

As for all previously described UniCAR/TM combinations, induction of tumor cell eradication via the UniCAR system was also strictly dependent on the presence of the novel bivalent α-EGFR-EGFR TM. Not unexpected, as also seen in all of our previous retargeting studies the degree of tumor cell lysis varied in a wide range depending on the chosen T cell donor.

Due to raising the number of binding sites, the resulting bivalent α-EGFR-EGFR TM shows (i) an increased apparent avidity in comparison to the affinity of the monovalent one, (ii) an improved killing efficacy and capability *in vitro* and *in vivo*, (iii) increased cytokine release of pro-inflammatory cytokines IFN-γ and TNF as well as growth-promoting cytokine IL-2 and (iv) an improved PET imaging contrast.

Obviously, improving the avidity of the α-EGFR TM increases the targeting capability: While the monovalent α-EGFR TM strongly binds only to tumor cells expressing high levels of EGFR, the bivalent α-EGFR-EGFR TM is able to efficiently bind to tumor cells with high, intermediate or even low EGFR surface expression levels. In line with these binding data, the monovalent α-EGFR TM stimulates UniCAR T cells only to attack tumor cells expressing high levels of EGFR, while the bivalent α-EGFR-EGFR TM engages UniCAR T cells even for an efficient lysis of cancer cells expressing low levels of EGFR. Nevertheless, it does not induce killing of MDA-MB-435S cells, whose number of EGFR molecules per cell is similar to healthy cells [44]. Hence, the risk for destruction of healthy tissues seems to be low.

As demonstrated by the TM titration experiments, 250-fold lower concentrations of the bivalent TM were able to activate UniCAR-armed T cells for tumor cell killing compared to the monovalent α-EGFR TM. Consequently, the three-fold increase of avidity shifted EC_50_ values from the nano- to the picomolar range.

In agreement with the improved EC_50_ value of the bivalent α-EGFR-EGFR TM, we could observe an increased anti-tumor effect in experimental mice. In our previous study, it was proven that the monovalent TM is able to mediate anti-tumor effects in a MRD mouse model [23]. Lowering the applied concentration of the monovalent construct ten times, resulted in loss of its functionality. On the contrary, under these limiting conditions the bivalent α-EGFR-EGFR TM is still capable of activating UniCAR T cells for tumor cell killing. Consequently, the application of the bivalent α-EGFR-EGFR TM in combination with the UniCAR system is favorable with regard to its therapeutic effects at low TM concentrations and low levels of target expression but at the risk of an increased cytokine release.

As recently published [23], the monovalent α-EGFR TM is mainly eliminated via the kidneys. In contrast, the bivalent α-EGFR-EGFR TM is eliminated via both, kidneys and liver. The altered elimination in combination with the improved avidity of the bivalent TM finally leads to an enhanced enrichment at the tumor site and thereby to an improved imaging contrast. Thus, the bivalent α-EGFR-EGFR TM gains favorable targeting and imaging properties at the cost of an enhanced risk of cytokine release.

In summary, the here presented data show that UniCAR T cell-mediated tumor cell killing not solely depends on the affinity between the UniCAR and the UniCAR epitope of TMs but also on the affinity of the TM to the respective TAA as well as on the density of the TAA on the tumor cells. Below a certain TM affinity or tumor antigen density redirected UniCAR-armed T cells will only induce suboptimal or even no cell lysis. On the one hand, this could be favorable to spare healthy tissues expressing levels of the TAA below this threshold. On the other hand, tumor cells which express the target at low levels could be able to escape killing. To circumvent the escape of such low target antigen expressing tumor cell variants, enhancing the affinity with e.g. bivalent or combinatorial TMs represents an attractive strategy. However, such enhanced TMs may show an enhanced risk for CRS. Thus, a two-step UniCAR therapy could be favorable: A UniCAR-based therapy may be started using a small TM such as the monovalent α-EGFR TM that may have a reduced risk of CRS and can rapidly be turned off in case severe CRS and/or tumor lysis syndrome occurs. Once the major tumor burden has been destroyed and the risk of these side effects are low, UniCAR-modified T cells may be armed with the more risky TM such as the α-EGFR-EGFR TM which, however, allows the killing of tumor cells expressing low levels of the TAA.

## MATERIALS AND METHODS

### Cell lines

The epidermoid carcinoma cell line A431, the squamous cell carcinoma cell line FaDu, the breast cancer cell line MDA-MB-435S as well as the CHO cell line were purchased from American Type Culture Collection and have not been further authenticated. The recombinant firefly luciferase-expressing target cell line A431-Luc and the recombinant PSCA-expressing cell line PC3-PSCA were generated via lentiviral transduction as described previously [45]. The CHO and the PC3-PSCA cell lines were cultured in complete RPMI 1640 medium [45]. FaDu cells and MDA-MB-435S cells were kept in complete DMEM medium [45] whereas the A431 and the A431-Luc carcinoma cell lines were grown in complete DMEM medium supplemented with 1 mM sodium pyruvate (Biochrom GmbH, Berlin, Germany). All cells were maintained at 37° C in a humidified atmosphere of 5% CO_2_.

### Construction, expression and purification of recombinant antibodies

Cloning of the monovalent α-EGFR TM into the lentiviral vector p6NST50, transduction of CHO wt cells, Ab purification and analysis (SDS-PAGE, immunoblotting) were described previously [23]. The novel bivalent α-EGFR-EGFR TM is based on a camelid Nb derived from the α-EGFR Ab clone 7C12 [43]. In a first step, the synthesized gene *Nhe*I-α-EGFR Nb-E5B9-α-EGFR Nb-*Mss*I was purchased from the company Eurofins Genomics (Ebersberg, Germany). To enable eukaryotic expression the open reading frame of the novel bivalent TM was cloned into the lentiviral vector p6NST50 as described previously [23], resulting in the vector p6NST50_α-EGFR-EGFR TM. After transducing the vector into CHO wt cells [46] the stably expressed TM was purified via Ni-NTA column [45, 47, 48] and subsequently analyzed by SDS-PAGE and immunoblotting as published before [46, 48, 49].

### High-performance liquid chromatography

For determination of the molecular weight and purity level of the purified α-EGFR TM, α-EGFR-EGFR TM and CHO wt supernatant, size exclusion high-performance liquid chromatography (SE-HPLC) was executed as described previously [23]. Therefore, a 15 µl sample containing 15 µg of the respective TM or 15 µl of CHO wt supernatant were applied into the HPLC system.

### Activation and inhibition of EGFR signaling by Nb-based TMs

In order to investigate the effects of EGFR-specific TMs on EGFR signaling, phosphorylation of the receptor was analyzed on the basis of a method published by Roovers *et al.* [50]. In brief, 1 x 10^5^ A431 tumor cells were cultured overnight in complete DMEM medium containing 0.1% FCS (Biochrom GmbH). The next day, cells were washed and incubated in the same medium containing 1% BSA and either 8 nM EGF (Gibco BRL, Eggenstein, Germany) or 1000 nM α-EGFR TM or 1000 nM α-EGFR-EGFR TM. After 15 min at 37° C, cells were placed on ice and harvested by scraping them off the plate in 50 µl RIPA buffer and 50 µl 2× Laemmli protein sample buffer. A431 cells were further lysed by using a QIAshredder homogenizer (Qiagen GmbH, Hilden, Germany) and by boiling the resulting lysate at 95° C. Analysis of proteins was performed by SDS-PAGE with subsequent immunoblotting as already published [46, 48, 49]. Phosphorylated EGFR (pEGFR) was detected by a mouse α-pEGFR (tyrosine 1068) mAb (Cell Signaling Technology, Danvers, MA, USA) and an alkaline phosphatase (AP)-conjugated α-mouse IgG (Dianova, Hamburg, Germany). Additionally, β-tubulin served as loading control. Therefore, the same blot was stained against β-tubulin using a monoclonal β-tubulin mAb (Thermofisher, Dreieich, Germany). In addition to agonistic effects, the potential of Nb-based TMs to inhibit EGF-induced signaling was investigated. For this purpose, A431 cells were incubated with a mixture of EGF (8 nM) and decreasing amounts of the respective TM (1000 nM, 100 nM, 10 nM, 1 nM). Afterwards, phosphorylation of EGFR was detected by immunoblotting.

### Flow cytometry analysis

To determine the expression level of EGFR on MDA-MB-435S, PC3-PSCA, FaDu and A431 cell lines, the cells were stained with the mouse α-EGFR IgG1 mAb (clone AY13; BioLegend, Fell, Germany). Quantification was performed using the QIFIKIT^®^ (Agilent Technologies, Böblingen, Germany) and the included fluorochrome-labeled α-mouse IgG mAb according to the manufacturer’s instructions. Binding properties of the novel α-EGFR-EGFR TM or the monovalent α-EGFR TM to different tumor cell lines was assessed by immunofluorescent staining as described before [45, 51]. As secondary Ab the mouse α-E5B9 IgG2a Ab and as tertiary Ab a Pacific Blue™-conjugated α-mouse-IgG (Fcγ) Ab (Life Technologies, Darmstadt, Germany) was used. Flow cytometry was performed with the MACSQuant^®^ Analyzer and the MACSQuantify^®^ software (Miltenyi Biotec GmbH, Bergisch Gladbach, Germany). K_d_ values were calculated as described [23].

### Isolation and lentiviral transduction of human T cells

Primary human T cells were isolated from peripheral blood mononuclear cells (PBMCs) out of buffy coats obtained from the German Red Cross (Dresden, Germany) with consent of the donors. The isolation steps and following cultivation of T cells in complete RPMI 1640 medium supplemented with 200 U/ml IL-2 (Proleukin^®^ S, Novartis Pharmaceuticals, Horsham, UK), 5 ng/ml IL-7 and 5 ng/ml IL-15 (ImmunoTools, Friesoythe, Germany) were performed as described elsewhere [45, 46]. Subsequently, T cells were transduced with the lentiviral vector encoding either the EGFP marker protein (vector control), the UniCAR construct containing a dual CD28/CD3ζ signaling domain (UniCAR 28/ζ) or the UniCAR construct lacking this domain (UniCAR stop) [22]. Production of lentiviral particles and transduction of human T cells was carried out as described previously [24, 52].

### Cytotoxicity assay

To analyze the TM-mediated killing of tumor cells standard chromium release assays were performed as published before [e.g. 45].

### Cytokine-release assay

For determination of IFN-γ, TNF and IL-2 concentrations in cell-free supernatants enzyme-linked immunosorbent assay (ELISA) was performed as described previously [45]. Therefore, 48 h after start of co-cultivation supernatants were collected and analyzed using OptEIA™ Human IFN-γ, OptEIA™ Human TNF and OptEIA™ Human IL-2 ELISA Kits (BD Biosciences, Heidelberg, Germany) according to the manufacturer’s instructions.

### Radiolabeling

The production of ^64^Cu was performed at Cyclone(R) 18/9 (Helmholtz-Zentrum Dresden-Rossendorf) in a ^64^Ni(p, n) ^64^Cu nuclear reaction with specific activities of 150–250 GBq/mmol Cu diluted in HCl (10 mM). For radiolabeling of the respective TM with ^64^Cu, the pH of the ^64^Cu solution was adjusted to pH 5.2 using NH_4_OH and 1.6 nmol of the respective TM were added. The respective mixtures were shaken at 37° C for 30 min. Then 1 µmol EDTA was added and the radiolabeled TM was separated by spin filtration with PBS. The labeling process was monitored using instant thin-layer chromatography (ITLC). After chelating, the reaction mixture was supplemented with EDTA, and the radiolabeling efficiency was determined using both ITLC and size-exclusion high-performance liquid chromatography (SE-HPLC). SDS-PAGE of the labeled conjugates, followed by silver staining and autoradiography was performed to further evaluate the TM-specific conjugation.

### Optical imaging, small animal PET imaging and biodistribution analysis of tumor xenograft models

Female NMRI-Foxn1^nu^/Foxn1^nu^ mice (JANVIER LABS, St. Berthevin, France) were kept at the Helmholtz-Zentrum Dresden-Rossendorf (HZDR) according to the guidelines of German Regulations for Animal Welfare. All animal experiments have been approved by the Landesdirektion Dresden (24-9165.40-4, 24.9168.21-4/2004-1).

For optical imaging of anti-tumor effects, 1.5 × 10^6^ A431 cells were investigated alone or together with 1.5 × 10^6^ human UniCAR 28/ζ-armed T cells in the presence or absence of 600 pmol of the α-EGFR TM or the α-EGFR-EGFR TM. Cell mixtures (100 µl/mouse) were subcutaneously injected into the right tight of eight-week-old experimental mice. To analyze the tumor growth general anesthesia was induced as published recently [23, 25]. After i.p. injection of luciferin (200 µl, 15 mg/ml) (Thermofisher, Dreieich, Germany) luminescence imaging and X-ray photography were performed using a dedicated small animal multimodal imaging system (Xtreme, Bruker, Germany) as described previously [24].

For PET imaging, immunodeficient mice, aged 5–8 weeks, were subcutaneously injected in the right hind flank with 1 x 10^6^ A431-Luc cells. After six to eight weeks, tumor size was measured as already described [23]. For pharmacological analyses, animals with 100–500 mm^3^ tumors were selected. After labeling of respective Ab constructs with ^64^Cu [23, 25], approximately 3.7 MBq [^64^Cu]Cu-α-EGFR-NODAGA TM or [^64^Cu]Cu-α-EGFR-EGFR-NODAGA TM were intravenously inoculated into a lateral tail vein of four A431-Luc tumor-bearing NMRI^nu/nu^ mice. Dynamic PET scans were acquired over 120 min using a small animal PET/CT scanner (NanoPET/CT, Mediso). In addition, a static scan was obtained 20 h after injection. Visualization was performed via InterView (Mediso) and ROVER software (ABX GmbH). After quantification, data were expressed as standardized uptake value (SUV), representing the activity concentration normalized to the body weight. SUV is defined as tissue concentration (MBq/ml) x body weight (g)/injected dose (MBq). The corresponding time activity curves (TAC) are based on the average ± SEM.

To analyze the tumor targeting and the biodistribution of the ^64^Cu-radiolabeled mono- and bivalent TM, four A431-Luc tumor-bearing NMRI^nu/nu^ mice were intravenously injected with approximately 0.5 MBq of the respective construct. After 2 h incubation time, mice were killed and selected organs, tissues as well as blood were taken and measured as already described [23]. Quantitative data were expressed as SUV.

### Statistical analysis

Statistical analysis of multiple experiments was performed with GraphPad Prism software version 6.0 (GraphPad Software Inc., La Jolla, CA, USA) using one-way ANOVA with posthoc Bonferroni Multiple Comparison or Tukeys test.

## SUPPLEMENTARY MATERIALS FIGURE


