# Supplementary material for: From mono- to bivalent: improving theranostic properties of target modules for redirection of UniCAR T cells against EGFR-expressing tumor cells in vitro and in vivo
Source: Oncotarget. 2018 May 22;9(39):25597–616. doi: 10.18632/oncotarget.25390 (PMC5986651; doi:10.18632/oncotarget.25390)
Supplement: Supplementary file 1 [file oncotarget-09-25597-s001.pdf]

From mono- to bivalent: improving theranostic properties of target modules for redirection of UniCAR T cells against EGFR-expressing tumor cells *in vitro* and *in vivo*

SUPPLEMENTARY MATERIALS

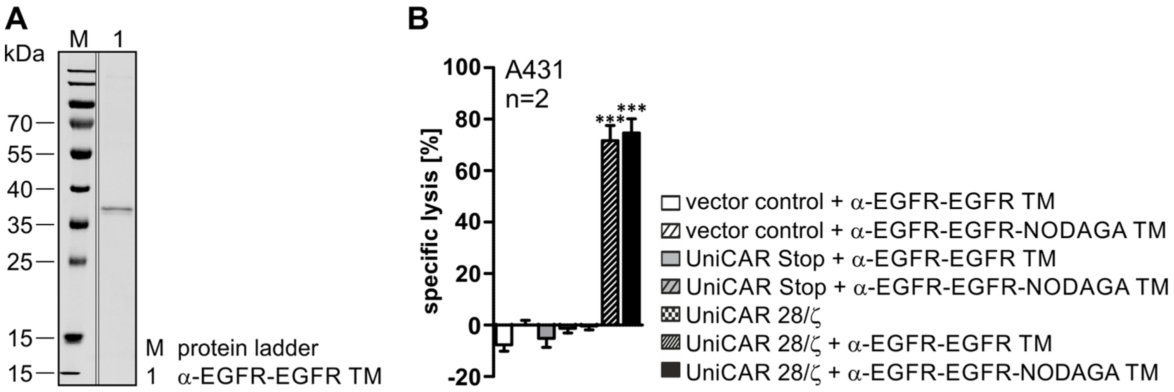

Supplementary Figure 1: Biochemical and functional characterization of PET grade bivalent α-EGFR-EGFR TM. (**A**) For radiolabeling the α-EGFR-EGFR TM contaminating HMW proteins were removed and the level of purity estimated by SDS-PAGE and staining with Coomassie Brilliant Blue G250. (**B**) After conjugation with the chelator NODAGA the α-EGFR-EGFR TM was functionally compared with the α-EGFR-EGFR-NODAGA TM. Therefore, genetically modified T cells were co-cultivated with ^{51}Cr-labeled A431 tumor cells in an effector to target cell ratio of 5:1 in the presence or absence of 50 nM of the α-EGFR-EGFR TM or the α-EGFR-EGFR-NODAGA TM for 48 h. As effector cells served T cells modified with either the vector control, the UniCAR Stop construct or the UniCAR 28/α construct. Mean specific lysis and SD for two independent T cell donors are shown ($^{***}p < 0.001$; with respect to controls: vector control or UniCAR Stop + TM and UniCAR 28/α; one-way ANOVA with Bonferroni multiple-comparison test).
